# Comparison of senescence-related changes between three- and two-dimensional cultured adipose-derived mesenchymal stem cells

**DOI:** 10.1186/s13287-020-01744-1

**Published:** 2020-06-09

**Authors:** Qiliang Yin, Na Xu, Dongsheng Xu, Mingxin Dong, Xiumin Shi, Yan Wang, Zhuo Hao, Shuangshuang Zhu, Donghai Zhao, Haofan Jin, Wensen Liu

**Affiliations:** 1 Cancer Center at the First Hospital of Jilin University, 1 Xinmin Street, Changchun, 130021 People’s Republic of China; 2 Jilin Medical University, Jilin, 132013 People’s Republic of China; 3 Institute of Military Veterinary Medicine, Academy of Military Medical Sciences, Zoonosis Prevention and Control Key Laboratory, Changchun, 130122 People’s Republic of China

**Keywords:** Adipose-derived mesenchymal stem cells, Three-dimensional culture, Senescence, Energy metabolism

## Abstract

**Background:**

Adipose-derived mesenchymal stem cells (ADMSCs) have attracted widespread interest as cell-based tissue repair systems. To obtain adequate quantities of ADMSCs for therapeutic applications, extensive in vitro expansion is required. However, under current two-dimensional (2D) approaches, ADMSCs rapidly undergo replicative senescence, and cell growth is impeded and stem cell properties are eliminated by mechanisms that are poorly understood. These issues limit the extensive applications of ADMSCs. In this study, we investigated senescence-related changes in mesenchymal stem cells (MSCs) isolated from human adipose tissue in 2D and three-dimensional (3D) cultures.

**Methods:**

We studied cell growth over a given period (21 days) to determine if modes of culture were associated with ADMSC senescence. ADMSCs were isolated from healthy females by liposuction surgery and then were grown in 2D and 3D cultures. The cell morphology was observed during cell culture. Every other time of culture, senescence-associated β-galactosidase (SA-β-gal) expression, cell viability, proliferation, and differentiation potential of ADMSCs from 2D and 3D cultures were detected. Also, senescence- and stemness-related gene expression, telomere length, telomerase activity, and energy metabolism of ADMSCs for different culture times were evaluated.

**Results:**

With long-term propagation, we observed significant changes in cell morphology, proliferation, differentiation abilities, and energy metabolism, which were associated with increases in SA-β-gal activity and decreases in telomere length and telomerase activity. Notably, when cultured in 3D, these changes were improved.

**Conclusions:**

Our results indicate that 3D culture is able to ameliorate senescence-related changes in ADMSCs.

## Background

Mesenchymal stem cells (MSCs) are pluripotent stem cells with the potential to self-replicate and multi-differentiate [1, 2]. Adipose tissue is an important source of MSCs [3]. Adipose-derived mesenchymal stem cells (ADMSCs) are harvested with low donor-site morbidity and are not associated with ethical issues; therefore, they represent promising candidates for various clinical applications, including tissue repair and regenerative medicine [2, 4]. In recent years, ADMSCs have been shown to promote revascularization, activate local stem cell niches, reduce oxidative stress, and modulate immune responses [3, 5]. However, therapies utilizing ADMSCs often require ex vivo expansion to generate large quantities of cells required for patients [6, 7]. Therefore, in vitro expansion is particularly important for ADMSCs.

Two-dimensional (2D) culture has been routinely used over the past several decades [8]. Typically, MSCs are expanded on stiff tissue culture-treated polystyrene, as a 2D monolayer [6]. However, growing cells in flat layers on plastic surfaces do not accurately mimic the natural in vivo cellular microenvironment, due to a lack of three-dimensional cues from the external media [9]. Moreover, the cellular microenvironment can seriously influence MSC characteristics and cause issues when transferring basic research to clinical settings [10]. Accumulating evidence indicates that extensive passaging of ADMSCs in 2D culture induces replicative senescence, resulting in cell cycle arrest, cell morphology and metabolic changes, and loss of differentiation potential [11–14]. Therefore, ADMSCs are prone to senescence and are difficult to maintain during long-term 2D expansion [15]. As a result, efforts have led to the development of novel approaches to recreate more physiologically relevant environments in the form of three-dimensional (3D) culture [16, 17].

3D culture is a simple and effective culture system, developed on the basis of a 2D monolayer, but with in vivo animal model characteristics [18, 19]. Currently, 3D culture systems are gaining interest with regard to recreating complex extracellular microenvironments (ECMs), thereby providing insights into conditions experienced by MSCs. When compared to 2D approaches, 3D culture creates an artificial ECM where cells grow or interact with their surroundings in three dimensions [8, 20]. In such cultures, there is increased MSC communication with neighboring cells, and cell-to-cell and cell-to-matrix connections are easily formed [21]. Reports from the literature suggest that 3D culture of human umbilical cord MSCs promotes cell yields, maintains stemness, and represents a promising strategy for cell expansion on industrial levels, with great potential for cell therapy and biotechnology [22–24]. Additionally, 3D microenvironments encourage MSC growth and differentiation into hepatocyte-like cells, in the presence of growth factors [25]. More importantly, the utilization of 3D culture techniques circumvents issues surrounding altered cellular properties of extensively expanded MSCs [7, 21]. In this work, we focus on the benefits of using 3D cultures and investigate ideal and optimized living environments for ADMSC growth in vitro.

## Methods

### Isolation and cultivation of human ADMSCs

Abdominal adipose tissue was obtained from five healthy females by liposuction surgery. Lipoaspirate MSCs were isolated and characterized as previously described [26]. Cells in culture were maintained at 37 °C with 5% CO_2_, until 80% cell confluence; then, cells were passaged on. ADMSCs from the third passage were plated in conventional 2D or 3D culture vessels. For 2D culture, cells from the third passage were cultured in six-well plates at 1 × 10^6^ cells/ml. Hydrogel (TheWell Bioscience, catalog no. TWG002, Shanghai, China), as a non-animal-derived polysaccharide hydrogel system that mimics the natural cellular microenvironment, is a new synthetic biomaterial for cell expansion in vitro and is the main constituent of 3D culture. The hydrogel was mixed with cell culture medium to form a hydrogel matrix. The dilution ratio was 1:1, i.e., hydrogel to PBS, 1:1, v/v. The hydrogel and ADMSCs were uniformly mixed and were seeded into a six-well plate at 1 × 10^6^ cells/ml as the 3D culture. Then, the cell culture medium (DAKEWE, EliteGro™-Adv, Beijing, China) was added to cover the hydrogel carefully. The medium was replaced every 3 days. When ADMSCs grew to 80% confluence, the cells were passaged at 1:2. It was a little different for the cell passage of 3D culture. Firstly, preheated 0.1× PBS and empty centrifuge tube in 37 °C water bath before taking out the cell culture plate from incubator. Discarded the medium covered with the top of the hydrogel, added 1 ml of preheated 0.1× PBS into each hole, and thoroughly mixed. Then, put the mixture into the preheated centrifuge tube and rinsed each hole with 1 ml preheated 0.1× PBS, and added preheated 0.1× PBS to 10 ml, thoroughly mixed. Finally, collected the cells after centrifuging at 1000 rpm for 5 min. ADMSCs were reseeded at six-well plate.

The first day (d 1) was defined when ADMSCs from the third passage were seeded into six-well plates, as described.

### Characterization of ADMSCs

Flow cytometry assessment was conducted to confirm the mesenchymal origin of cells. Third passage cells were resuspended following digestion with 0.125% trypsin. A minimum of 1 × 10^5^ cells/ml were collected from six-well plates. Rat monoclonal anti-human antibodies were used at a dilution of 1:150 for all cell surface markers. MSCs were incubated with phycoerythrin (PE)-coupled antibodies, CD34 (sc-7324; Santa Cruz) and CD45 (554,878; BD Biosciences), and fluorescein isothiocyanate (FITC)-coupled antibodies, CD44 (550,974; BD Biosciences) and CD105 (BD Biosciences), in the dark at room temperature for 30 min. IgG1-PE and IgG1-FITC were used as isotype controls. Cell data were analyzed using the Paint-A-Gate Pro™ software.

### Senescence-associated (SA)-β-galactosidase (gal) assay

ADMSCs from 2D and 3D cultures at 3 days, 7 days, 14 days, and 21 days were seeded in six-well plates at 1 × 10^5^ cells/well overnight. The next day, cells were fixed for 30 min at room temperature in 4% formaldehyde and washed twice in PBS (pH 7.3). Then, ADMSCs were incubated overnight at 37 °C with freshly prepared SA-β-gal stain solution (C0602, Beyotime, Shanghai, China) following the manufacturer’s instructions. At least 400 cells were observed in randomly chosen, non-overlapping fields by three independent observers to quantify SA-β-gal expression. Positive cells were stained blue and counted in three randomly selected fields under the microscope (XPF-550C, caikon, Shanghai, China). The experiment was performed three times, and the mean percentage of cells expressing SA-β-gal was calculated.

### Cell viability

ADMSC viability was determined using Muse Count & Viability Assay in the Muse Cell Analyzer (Luminex, USA) according to the manufacturer’s instructions. Cells (1 × 10^6^ cells/well) were harvested from both 2D and 3D cultures. They were then resuspended in medium to achieve a cell density of 2 × 10^5^ cells/ml, from which 50 μl cells and 450 μl Muse™ Count & Viability reagent were added to a tube, and incubated for 5 min in the dark at room temperature. Cell viability was evaluated by miniaturized fluorescence detection and microcapillary cytometry with Muse Cell Analyzer. In addition, we counted the cells in 2D and 3D cultures at every measured time point by Muse Cell Analyzer.

### Stain of living cells

ADMSCs from 2D and 3D cultures at 3 days, 7 days, 14 days, and 21 days were stained by HCS NuclearMask™ Blue Stain (H10325, Invitrogen, Shanghai, China) according to the manufacturer’s instructions. The cells were pulsed with 100 μl staining solution (1:2000) for 30 min at room temperature. After the incubation, the stained cells were analyzed by fluorescence microscopy (IX71, OLYMPUS, Xiameng, China).

### Adipo- and osteogenic differentiation of ADMSCs

ADMSCs from 3 days, 7 days, 14 days, and 21 days were induced to undergo adipogenic and osteogenic differentiation, to identify cell capacity for differentiation in 2D and 3D cultures. For adipogenic differentiation, ADMSCs were seeded in 24-well plates at a density of 1 × 10^5^ cells/well. MSCgo™ adipogenic differentiation medium (catalog no. 05412, STEMCELL, USA) was used to induce adipogenic differentiation after cells reached 95% confluence. Induction/maintenance media were replaced for cycles of 3 days/1 day, respectively. Differentiated cells were assessed by staining intracellular lipid droplets with Oil Red O (MC37A0-1.4, VivaCell BIOSCIENCES, Shanghai, China) after 21 days in adipogenesis induction medium. For osteogenic differentiation, a similar process was adopted. MSCgo™ osteogenic differentiation medium (catalog no. 05465, STEMCELL, USA) induced osteogenic differentiation when cells reached 95% confluence. Differentiated cells were stained using Alizarin Red S (MC37C0-1.4, VivaCell BIOSCIENCES, Shanghai, China) staining of accumulated calcium deposits, after 28 days of differentiation. At least 300 cells were observed in randomly chosen, non-overlapping fields by three independent observers to quantify cell differentiation capacity. Positive cells were counted in three randomly selected fields under the microscope (XPF-550C, caikon, Shanghai, China).

### Real-time fluorescence quantitative polymerase chain reaction (RT-qPCR)

Total RNA was isolated from ADMSCs using TRIzol reagent (Invitrogen, USA) following the manufacturer’s instructions. cDNA was prepared by reverse transcription using the PrimeScript™ RT-PCR Kit (TaKaRa, Japan). Next, mRNA levels were quantified for aging-related genes (p16, p21, p53) and stemness-related genes (Sox2, Oct4, Nanog, c-myc) by RT-qPCR on an ABI Prism7900 Detector (Applied Biosystems, USA), using SYBR Premix Ex Taq™. β-actin was used as a reference gene. Each experimental group was analyzed in triplicate. mRNA expression was calculated using the 2^−ΔΔCt^ method. Primer sequences for RT-qPCR are shown in Table 1.

**Table 1:** Primers used for RT-qPCR

Gene	Primer sequence (5′–3′)	Product size (bp)
β-actin	Forward: CATGTACGTTGCTATCCAGGC	250
	Reverse: CTCCTTAATGTCACGCACGAT	
P16	Forward: ATCATCAGTCACCGAAGG	369
	Reverse: TCAAGAGAAGCCAGTAACC	
P21	Forward: CATCTTCTGCCTTAGTCTCA	163
	Reverse: CACTCTTAGGAACCTCTCATT	
P53	Forward: CGGACGATATTGAACAATGG	158
	Reverse: GGAAGGGACAGAAGATGAC	
SOX2	Forward: GCCGAGTGGAAACTTTTGTCG	155
	Reverse: GGCAGCGTGTACTTATCCTTCT	
OCT4	Forward: CTGGGTTGATCCTCGGACCT	243
	Reverse: CCATCGGAGTTGCTCTCCA	
Nanog	Forward: TCTATAACTGTGGAGAGGAATC	122
	Reverse: GGTCTGCTGTATTACATTAAGG	
c-myc	Forward: GTCAAGAGGCGAACACACAAC	162
	Reverse: TTGGACGGACAGGATGTATGC	
TERT	Forward: AAATGCGGCCCCTGTTTCT	76
	Reverse: CAGTGCGTCTTGAGGAGCA	

### Telomere length and activity assay

Genomic DNA (gDNA) was isolated from ADMSCs and used as a template for RT-qPCR. The relative telomere length of ADMSCs from 3 days, 7 days, 14 days, and 21 days was assayed using the relative human telomere length quantification RT-qPCR assay kit (catalog no. 8908, ScienCell, USA) following the manufacturer’s instructions. Data were analyzed using the comparative quantification cycle value (∆∆Cq) method. Telomeres are maintained by telomerase, which comprises telomerase reverse transcriptase (TERT) and telomerase RNA component (TERC) [27]; thus, TERT expression is consistent with telomerase activity. The RT-qPCR assay indirectly reflects telomerase activity by detecting TERT mRNA expression levels. The procedure was identical to the above.

### Relative mitochondrial DNA copy number quantification

Mitochondrial DNA (mtDNA) from ADMSCs was used as a template for RT-qPCR. The relative mtDNA copy number of ADMSCs from different time points was determined using the relative human mtDNA copy number quantification RT-qPCR assay kit (catalog no. 8938, ScienCell, USA) following the manufacturer’s instructions. Data were analyzed using the comparative ∆∆Cq method.

### Cellular energy metabolism studies

The extracellular acidification rate (ECAR) and oxygen consumption rate (OCR) of cells were detected using the XF96 extracellular flux analyzer (Seahorse Bioscience; North Billerica, MA, USA) following the manufacturer’s instructions. ADMSCs were seeded at 5 × 10^3^ cells/well in a 96-well Seahorse culture plate (Seahorse Bioscience, North Billerica, MA, USA), before conducting the experiment. For the ECAR assay, studies were performed in un-buffered DMEM (catalog no. 11965092, Gibco™, USA), pH 7.3 at 37 °C. Glucose (8 mM), oligomycin A (oligo; an ATP synthase inhibitor, 0.8 μM), and 2-deoxyglucose (2-DG; inhibitor of glycolysis; 80 mM) were added to different ports of the Seahorse cartridge. For OCR assays, analyses were conducted in a medium consisting of 20 mM glucose and 1.8 mM sodium pyruvate in un-buffered DMEM, pH 7.3, at 37 °C. Oligomycin A (1 μM), carbonyl cyanide m-chlorophenylhydrazone (FCCP; a mitochondrial uncoupler; 400 nM), rotenone (complex I inhibitor; 0.8 μM), and antimycin A (complex III inhibitor; 0.8 μM) were added to different ports of the Seahorse cartridge. Each experimental group was assayed with four to five replicates in each analysis. ECAR and OCR data were normalized to cell numbers, as detected by CellTiter-Glo analysis (Promega, USA) at assay end.

### Statistical data analysis

The numbers of repeats were at least 3 times for each experiment at both technical repeats and biological replicates. Numerical data were reported using means ± standard deviation (SD). Data analyses were performed using paired *t* tests with the GraphPad Prism 7 software. Statistical differences were assessed at *p* < 0.05, *p* < 0.01, and *p* < 0.001.

## Results

### Morphological characteristics of ADMSCs

Primary ADMSCs from culture are shown in Additional file 1: Fig. S1. These cells exhibited fibroblast-like, spindle-shaped morphology; were spiral-shaped; and were in alignment.

ADMSCs from the third passage were characterized by flow cytometry, indicating the presence of CD34- and CD45-negative (0.89%) surface markers (Additional file 2: Fig. S2a) and CD44- and CD105-positive (99.49%) surface markers (Additional file 2: Fig. S2b).

**Fig. 1:**
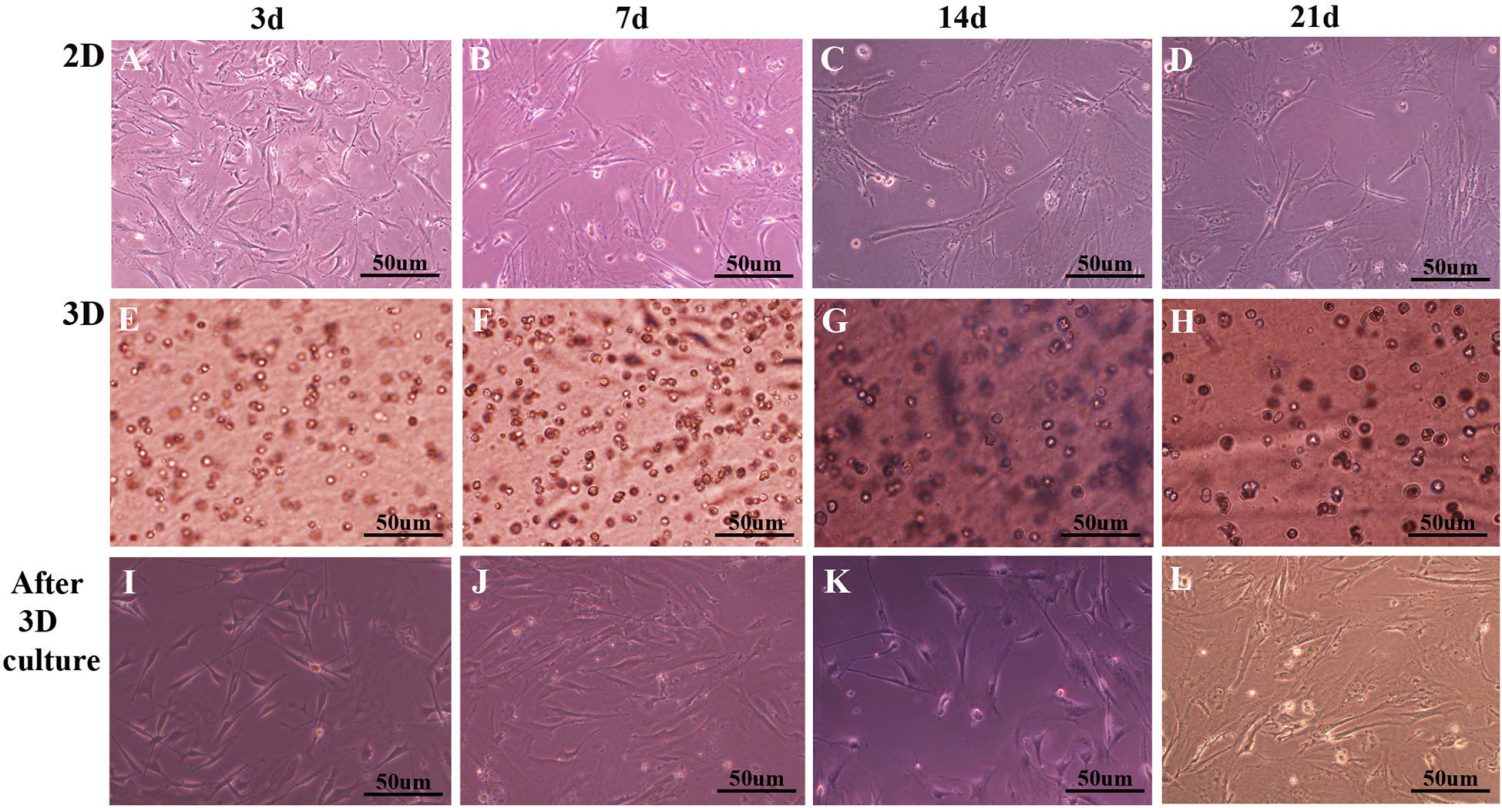
ADMSCs from the third passage were cultured in 2D and 3D cultures. **a**, **b** At 3 days and 7 days, cells were relatively homogeneous; they had characteristic spindle shapes and had smooth cell surfaces. **c** At 14 days, cells still maintained the characteristic MSC shape, but some cells appeared to have pseudopod-like structures and were longer and flatter. **d** At 21 days, cell shape was flat, and almost all ADMSCs lost their MSC shape; cells were focally aggregated and exhibited a “fried egg” morphology. **e**–**h** For 3D culture**,** most cells were round in shape and the cell density gradually increased. **g**, **h** At 14 days and 21 days, some cells appeared to adhere to the vessel wall. **i**–**l** Cells re-adhered to the vessel wall after 3D culture for 3 days, 7 days, 14 days, and 21 days, respectively. The cell shape gradually became longer and flatter, but most cells maintained elongated spindle shapes and had smooth cell surfaces. Cells never lost their characteristic MSC shape. The experiment was repeated three times

As described, ADMSCs from the third passage were plated in 2D and 3D cultures (Additional file 3: Fig. S3) and photographed at 3 days, 7 days, 14 days, and 21 days (Fig. 1). Cell morphology varied with different time points and culture modes. In 2D culture, cells showed a fibroblast-like morphology, were spindle-shaped, and were in alignment. At 3 days and 7 days, they were relatively homogeneous; cells had the characteristic spindle shape and the cell surface appeared smooth (Fig. 1a, b). At 14 days, 2D cultured cells still maintained the characteristic MSC shape; however, some cells displayed pseudopod-like structures, i.e., they were longer and flatter (Fig. 1c). Unlike 3 days and 7 days, cell shape at 21 days was flat, and almost all ADMSCs had lost their MSC shape, i.e., cells were focally aggregated and exhibited a “fried egg” morphology (Fig. 1d).

In contrast, there were no morphological variations in 3D culture, ADMSCs grew in a hydrogel suspension, and most cells were round in shape, with a gradually decreasing cell density in relation to time points (Fig. 1e–h).

In addition, ADMSCs were retrieved and re-cultured in six-well plates, without hydrogel after 3D culture for 3 days, 7 days, 14 days, and 21 days. Although cell shape gradually became longer and flatter as time progressed, most cells maintained elongated spindle shapes and smooth cell surfaces. More importantly, cells never lost their characteristic MSC shape (Fig. 1i–l).

### Evaluating senescence-associated (SA) β-galactosidase (gal) expression

**Fig. 2:**
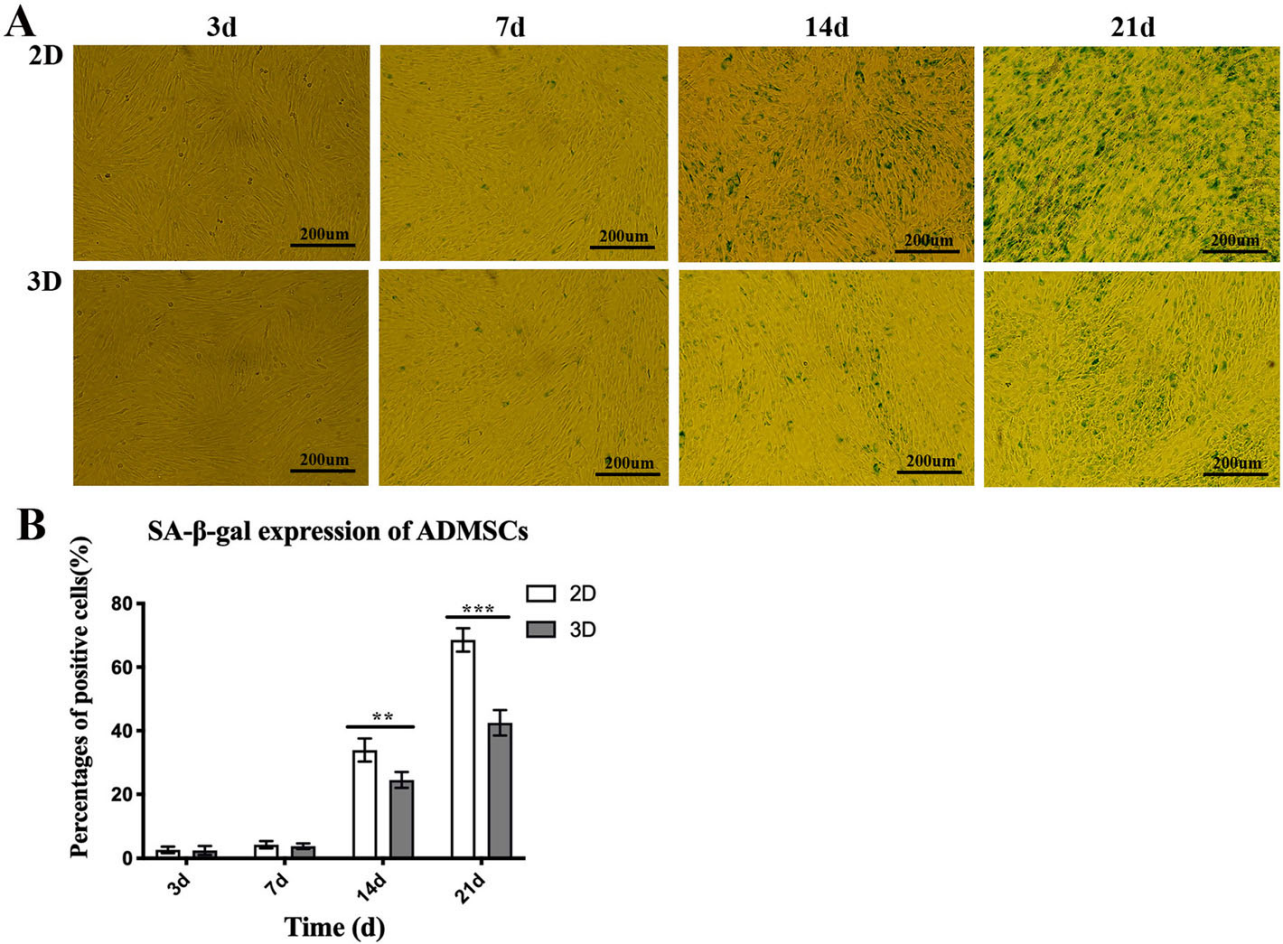
The expression of senescence-associated β-galactosidase in 2D and 3D ADMSC cultures. **a**, **b** Little or no SA-β-gal expression was detected at 3 days and 7 days in either cultures, but increased expression was observed during progressive cultivation with time. Blue ADMSCs in 2D culture were statistically higher than 3D cultures at 14 days. At 21 days, most ADMSCs were stained blue in 2D culture, but limited cells were stained blue in 3D culture at the same time period. Data represented as average ± SD from *n* = 3 experiments, **p < 0.01, ***p < 0.001

ADMSCs from 2D and 3D cultures were stained with SA-β-gal. Aging cells stained blue reflected SA-β-gal expression. As anticipated, little or no expression was observed at 3 days and 7 days in 2D and 3D cultures, but subsequent increases in expression were observed with cultivation times. While cell senescence was more visible in 2D cells when compared with 3D cells at 14 days and 21 days (Fig. 2a), blue ADMSCs in 2D culture were significantly increased when compared to 3D culture at 14 days (− 9.417 ± 0.651). Strikingly, most ADMSCs were stained blue at 21 days in 2D cultures, but limited cell densities were stained blue in 3D culture (26.08 ± 0.363), at the same time period (Fig. 2b).

### ADMSC viability

**Fig. 3:**
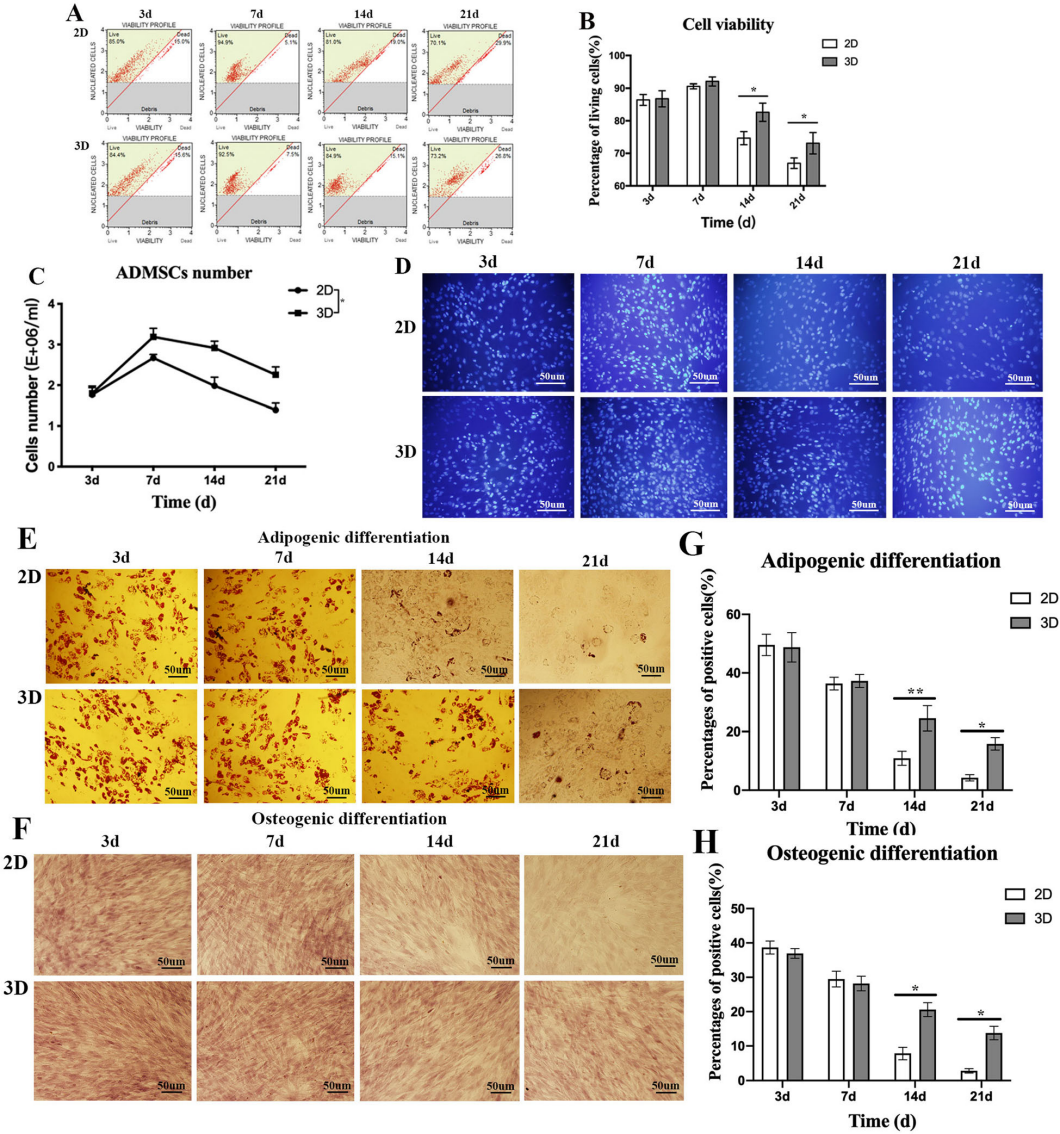
Cell viability of ADMSCs in 2D and 3D cultures at different time points. **a** ADMSC viability was comparable for both culture conditions up to 7 days. After this period, viability gradually decreased with time and was significantly decreased in 2D cultures when compared to 3D. **b** Cell viability in 3D cultures was significantly higher than 2D cultures at 14 days and 21 days. **c** The cell number in 2D and 3D cultures at every measured time point was counted. The cell number was significantly increased within 7 days in 2D and 3D cultures, and the population doubling time was 6 days and 4 days respectively; subsequently, the number of cells decreased rapidly, especially in 2D culture. Obviously, the cell number in 3D cultures was significantly greater than that in 2D. **d** Analysis of ADMSC staining showed that the number of living cells was basically in accordance with the result of cell viability. **e**–**h** Differences in adipo- and osteogenic capacity of ADMSCs in 2D and 3D cultures at 3 days, 7 days, 14 days, and 21 days. Adipo- and osteogenic differentiation potential of ADMSCs were similar at 3 days and 7 days, for both culture conditions. A significant age-related decline was observed in differentiation capacities at 14 days and 21 days in 2D cultures when compared with 3D. Cells practically lost all osteogenic differentiation in 2D cultures at 21 days. Data represented as average ± SD from *n* = 3 experiments, *p < 0.05, **p < 0.01

ADMSC viability was assessed in 2D and 3D cultures at different time points (Fig. 3a). As expected, ADMSC viability was comparable at 3 days and 7 days under both culture conditions, and the highest viability appeared at 7 days. However, viability decrease was observed at 14 days and 21 days and was significantly decreased in 2D cultures when compared with 3D. Significantly, cell viability in 3D culture was greater than in 2D cultures at 14 days (7.933 ± 1.281) and 21 days (6.133 ± 1.255) (Fig. 3b). Meanwhile, ADMSC number in 2D and 3D cultures at every measured time point was counted (Fig. 3c). Cell number was significantly increased within 7 days in 2D and 3D cultures, and the population doubling time was 6 days and 4 days, respectively; subsequently, the number of cells decreased rapidly, especially in 2D culture. Obviously, the cell number in 3D cultures was significantly greater than that in 2D (0.586 ± 0.411). Additionally, analysis of ADMSC staining showed that the number of living cells in 2D and 3D cultures was basically in accordance with the result of cell viability (Fig. 3d).

### Adipo- and osteogenic differentiation of ADMSCs

Differences in adipo- and osteogenic capacities of ADMSCs in 2D and 3D cultures over the time points 3 days, 7 days, 14 days, and 21 days were analyzed (Fig. 3e, f). Whether in 2D or 3D culture, the adipo- and osteogenic differentiation potential of ADMSCs were similar at 3 days and 7 days. However, an obvious age-related decline was observed in adipo- and osteogenic differentiation capacity for ADMSCs at 14 days (13.58 ± 2.184 and 12.75 ± 3.031, respectively) and 21 days (11.58 ± 2.309 and 10.95 ± 2.282, respectively) (Fig. 3g, h) in 2D cultures. Notably, in this culture, ADMSCs nearly lost osteogenic differentiation at 21 days (Fig. 3h).

### Evaluating aging- and stemness-related gene expression

**Fig. 4:**
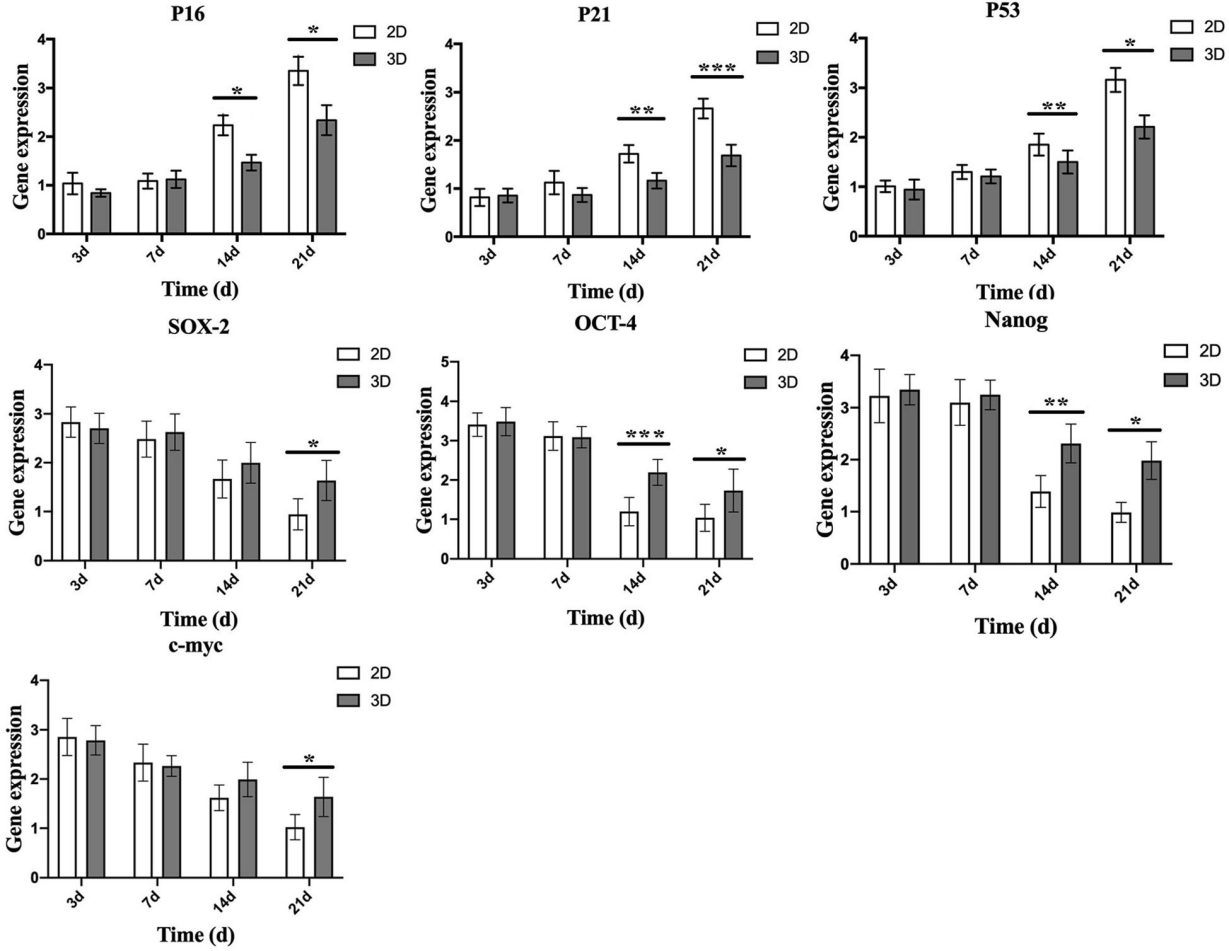
The expression of aging-related genes, p16, p21, and p53, gradually increased with culture time. The expression of all three genes in ADMSCs in 2D culture was significantly higher than 3D at 14 days and 21 days. In contrast, the expression levels of stemness-related genes, Sox2, Oct4, Nanog, and c-myc, exhibited a decreasing tendency. Moreover, Oct4 and Nanog expression exhibited a more significant reduction in 2D culture than 3D cultures at 14 days and 21 days; besides, Sox2 and c-myc expression were significantly decreased in 2D cultures at 21 days. Data represented as average ± SD from *n* = 3 experiments, *p < 0.05, **p < 0.01, ***p < 0.001

Changes in aging- and stemness-related gene expression were evaluated by quantifying mRNA levels in ADMSCs from both culture conditions. As expected, the expression of age-related genes p16, p21, and p53 was gradually increased with culture time; furthermore, gene expression in ADMSCs from 2D culture were significantly higher than those from 3D cultures at 14 days (− 0.767 ± 0.169, − 0.559 ± 0.019, and 0.351 ± 0.023, respectively) and 21 days (− 1.009 ± 0.178, − 0.975 ± 0.014, and − 0.947 ± 0.203, respectively). Conversely, the expression levels of stemness-related genes, Sox2, Oct4, Nanog, and c-myc, exhibited a decreasing tendency with long-term expansion. Moreover, Oct4 and Nanog expression exhibited a more significant reduction in 2D culture than in 3D cultures at 14 days (0.998 ± 0.021 and 0.921 ± 0.068, respectively) and 21 days (0.695 ± 0.201 and 0.997 ± 0.307, respectively); besides, Sox2 and c-myc expression were significantly decreased in 2D cultures at 21 days (0.693 ± 0.125 and 0.616 ± 0.143, respectively) (Fig. 4).

### Evaluation of relative telomere length, telomerase activity, and relative mitochondrial DNA copy number

**Fig. 5:**
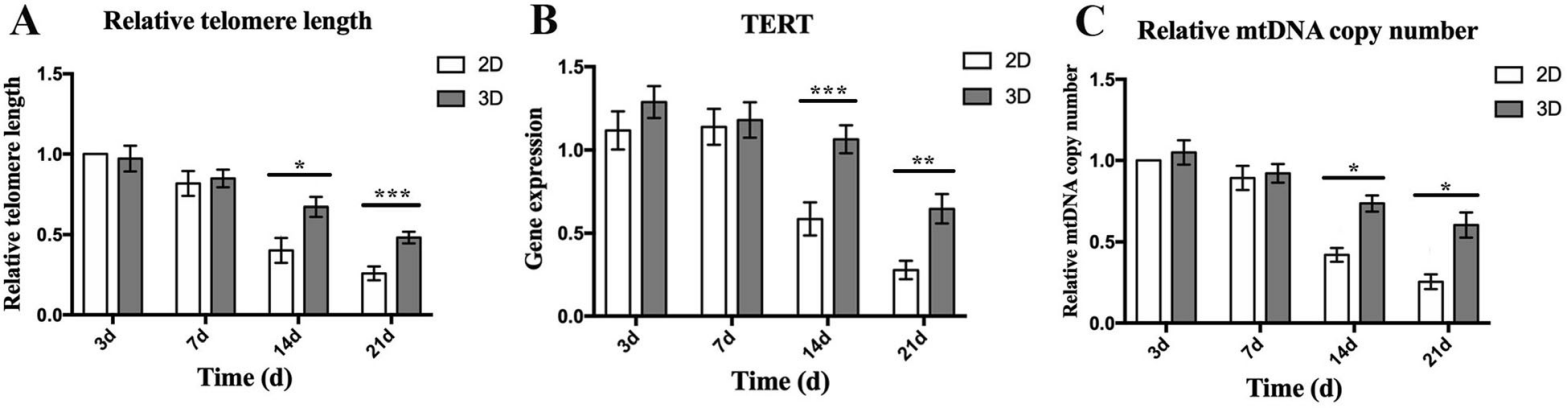
**a** Telomere length in ADMSCs gradually shortened during cell expansion in both 2D and 3D cultures. But telomere length shortened significantly in 2D cultures when compared to 3D, especially at 14 days and 21 days. Moreover, the shortening tendency in telomere length of ADMSCs in 3D cultures was steadier. **b** TERT expression revealed that changes in telomerase activity agreed with relative telomere length in ADMSCs for both cultures. **c** Relative mtDNA copy numbers in ADMSCs were gradually decreased, while mtDNA copy numbers in 3D cultures were significantly higher when compared with 2D cultures at 14 days and 21 days. Data represented as average ± SD from *n* = 3 experiments, *p < 0.05, **p < 0.01, ***p < 0.001

We identified that telomere length in ADMSCs gradually shortened during cell expansion in both 2D and 3D cultures. However, telomere length shortened significantly more in 2D culture when compared to 3D, especially for 14 days (0.274 ± 0.047) and 21 days (0.223 ± 0.0052). It seemed that the decline fluctuation of telomere length was steadier in 3D culture (Fig. 5a). In addition, TERT expression revealed that changes in telomerase activity agreed with relative telomere lengths in ADMSCs (Fig. 5b). Similarly, relative mtDNA copy numbers of ADMSCs in 2D and 3D cultures were gradually decreased, but mtDNA copy numbers in 3D culture were significantly higher when compared with 2D cultures at 14 days (0.315 ± 0.043) and 21 days (0.349 ± 0.055) (Fig. 5c).

### Changes in energy metabolism in ADMSCs

**Fig. 6:**
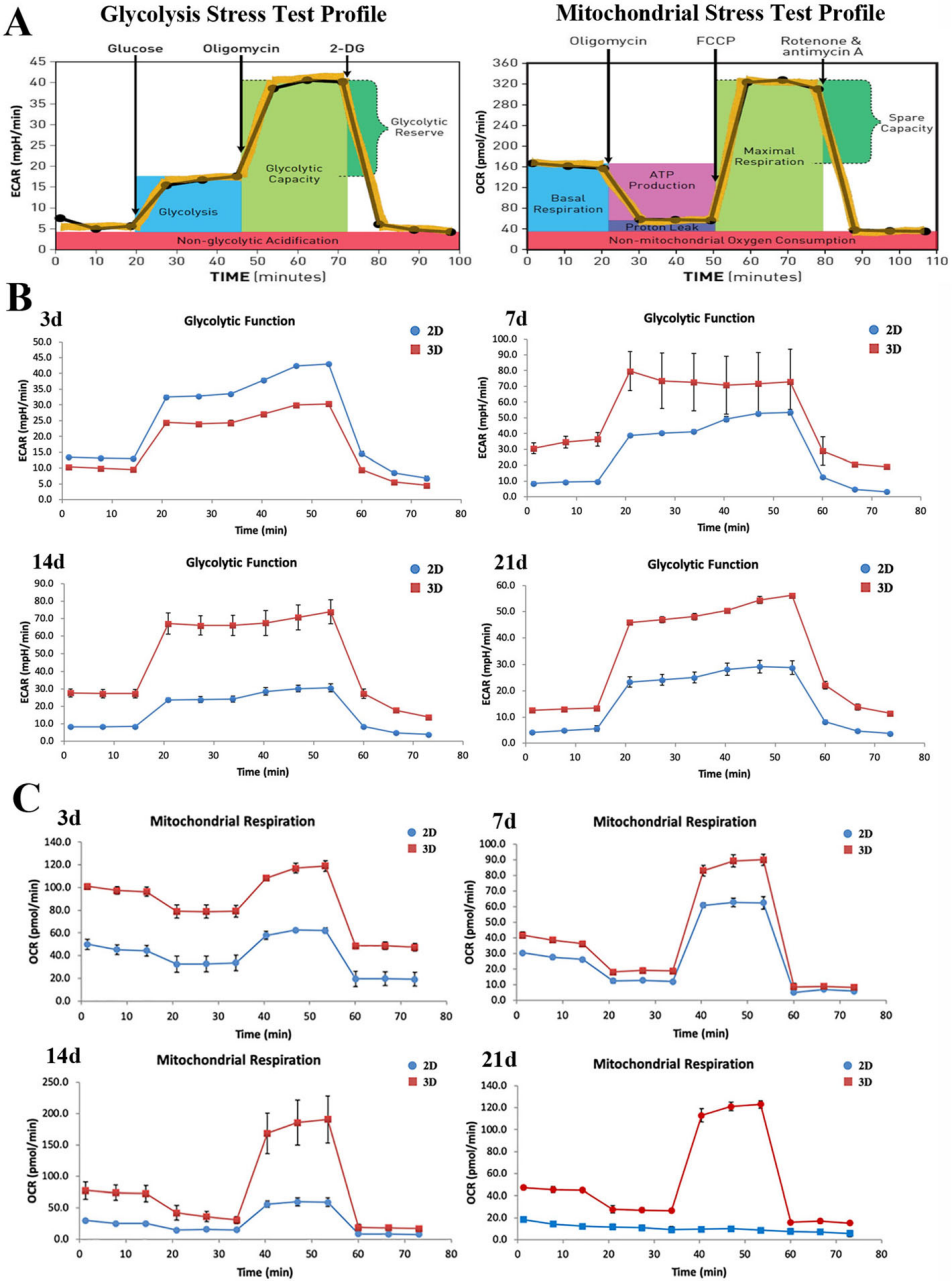
Change in glycolysis and mitochondrial respiration levels in ADMSCs. **a** Glycolysis and mitochondrial stress tests. **b** ECAR levels in ADMSCs in 3D cultures were higher than 2D cultures, at 7 days, 14 days, and 21 days, but lower at 3 days. **c** OCR levels in ADMSCs in 3D cultures were higher when compared with 2D cultures at each time point. The experiment was repeated five times

We analyzed the effects of 3D and 2D culture on ADMSC energy metabolism (Fig. 6a). It is generally accepted that glycolysis and mitochondrial respiration are two major energy production pathways in cells [28, 29]. Through glycolysis, ADMSCs utilize glucose to generate lactate; thus, if cells are compromised, increased lactate levels could create an acidic environment, as assessed by ECAR [30], and increased OCR is an indicator of mitochondrial respiration. We observed that ECAR levels in ADMSCs in 3D cultures were higher than in 2D cultures at 7 days, 14 days, and 21 days (Fig. 6b), but slightly less at 3 days, as ECAR levels showed little differences between 2D and 3D cultures at this early stage. OCR levels of ADMSCs in 3D culture were higher when compared with 2D culture, at each time point (Fig. 6c). Taken together, the 3D culture of ADMSCs appeared to induce positive regulation of glycolysis and mitochondrial respiration, i.e., 3D cultures sustained energy metabolism stability in ADMSCs.

## Discussion

Human ADMSCs are ideal candidates for diverse regenerative medicine approaches and tissue engineering strategies [31, 32]. However, it is interesting that therapies requiring ADMSCs often require ex vivo expansion approaches to generate the large numbers of cells required for patients and to overcome cell senescence limitations [6, 33]. It is widely recognized that 3D cultures create a pragmatic ECM and mimic in vivo development [20, 34]. Therefore, 3D culture of MSCs could increase cell yields, enhance differentiation, maintain stemness, and provide promising strategies for MSC expansion on an industrial scale, with great potential for cell therapy and biotechnology [20, 22].

In terms of morphological and biochemical characteristics, in vitro MSC senescence is characterized by an enlarged flat cellular morphology; an increased SA-β-gal activity; an increased expression of p16, p21, and p53 [12, 35]; and a loss of stem cell properties [36]. Studies have reported that MSCs enter replicative senescence after extensive culture, leading to morphological and functional cellular changes [23, 37]. Cell senescence is a major factor that affects the proliferation and multi-lineage potential of MSCs [23, 38]. Here, we discovered that proliferation and adipogenic and osteogenic differentiation in ADMSCs steadily declined following extensive expansion, but notably, the decline in 2D culture was greater than 3D. In addition, 3D ADMSCs at different culture times were re-adhering in vessels, without hydrogel; we observed that these cells were morphologically younger when compared with 2D culture cells, at the same time period; and more importantly, SA-β-gal expression in these cells (3D) was lower. These observations agreed with the literature showing that significant changes in cell morphology were associated with increased SA-β-gal expression [11]. Such phenomenon has been rarely reported, but our data provides critical evidence that 3D culturing exerts positive effects on senescence-associated changes. Interestingly, recent evidence has indicated that even a short 3D culture of 72 h altered extensively expanded MSC characteristics [7].

Telomere length and telomerase activity represent cell senescence at the cellular level [39]. Moreover, cell lifespan is directly proportional to telomere length [40]. Thus, telomere shortening more than likely functions as a mechanism implicated in cellular senescence [39, 41]. Reports have identified a direct correlation between telomerase activity and stem cell function [37], with telomere attrition contributing to aging [42]. Furthermore, studies have demonstrated that the relative telomere length of ADMSCs declines with long-term passaging [38]. Our study suggests that telomere length and telomerase activity of ADMSCs decline with aging, but may be improved by 3D culture, further confirming that 3D culturing exerts positive effects in delaying senescence in ADMSCs.

Although MSC aging mechanisms during long-term expansion remain ill-defined, it has been shown that in addition to telomere shortening and telomerase activity reduction inducing senescence, oxygen free radical generation and altered mitochondrial function may have plausible roles in aging [43]. Additionally, previous studies have demonstrated that senescence is associated with metabolic changes in the oxidative state of the cell and that this process is linked to glycolytic ability and mitochondrial function [11–13], suggesting associations between changes in energy metabolism and senescence. The metabolic energy changes observed in ADMSCs (in 2D and 3D cultures) in this study reveal that ECAR and OCR levels vary greatly between dimensional cultures, over different cultivation periods. Overall, ECAR and OCR levels were higher in 3D cultures, suggesting that this approach had positive effects on glycolytic function and mitochondrial respiration in ADMSCs. Up to now, ADMSC energy metabolism research is in its infancy, and relationships between energy metabolism changes and ADMSCs senescence had rarely been reported. Accordingly, this study has preliminarily confirmed that 3D culturing slows ADMSC senescence, by improving mitochondrial function and energy metabolism.

Indeed, some reports have indicated a direct relationship between mitochondrial dysfunction and stem cell aging [28, 44]. In several cell systems, mitochondrial dysfunction leads to respiratory chain dysfunction, which may be the result of mutation accumulation in mtDNA, in line with aging [45]. A critical mechanism underlying mitochondrial dysfunction is the expansion of mutations and deletions in mtDNA [29]. Additionally, these accumulated aging-related mutations and deletions lead to decreased mtDNA copy numbers [29, 45]. This study has shown that 3D culturing protects mtDNA in ADMSCs from aging-related impairments, suggesting that 3D cultures improve aging-related mitochondrial dysfunction.

Nevertheless, this study has some limitations. Firstly, some data did not reach statistical significance, which may be the result of lack of enough cell culture time in vitro. Similarly, we did not assess chondrogenic differentiation in ADMSCs, since the cells need to form spheroids during chondrogenic differentiation; the diversity in capability of sphere-forming of ADMSCs in 2D and 3D cultures has a great influence on chondrogenic differentiation; thus, it is difficult to evaluate chondrogenic differentiation ability of the ADMSCs. Finally, we revealed factors that impeded ADMSC growth in vitro, but an in-depth mechanistic exploration of factors that induce cellular senescence is required in future research.

## Conclusions

Taken together, this study confirms that 3D culturing relieves senescence-related changes in ADMSCs and highlights the importance of developing 3D culture approaches for sustained and healthy MSC growth. There is no doubt that 3D culturing will contribute to effective ADMSC preparations for cellular therapy, and importantly, our findings lay the path for future ADMSC applications in biomedical research. However, further investigations will be required to fully evaluate the effectiveness and safety of ADMSCs in 3D culture for future in vivo therapies.

## Supplementary information

**Additional file 1: Fig. S1.** ADMSCs exhibit fibroblast-like, spindle-shaped morphology, were spiral shaped and in alignment.

**Additional file 2: Fig. S2.** Flow cytometry analysis showing the presence of CD34 and CD45 negative (0.89%) surface markers **(a)**, and CD44 and CD105 positive (99.49%) surface markers **(b)**.

**Additional file 3: Fig. S3.** Model graph of 3D hydrogel culture.

## Data Availability

The datasets used and/or analyzed during the current study available from the corresponding author on reasonable request.

## Abbreviations

ADMSCs: Adipose-derived mesenchymal stem cells
2D: Two-dimensional
MSCs: Mesenchymal stem cells
3D: Three-dimensional
SA-β-gal: Senescence-associated β-galactosidase
ECM: Extracellular microenvironment
RT-qPCR: Real-time fluorescence quantitative polymerase chain reaction
gDNA: Genomic DNA
TERT: Telomerase reverse transcriptase
TERC: Telomerase RNA component
mtDNA: Mitochondrial DNA
ECAR: The extracellular acidification rate
OCR: Oxygen consumption rate
