# Supplementary figures and images for: Comparison of senescence-related changes between three- and two-dimensional cultured adipose-derived mesenchymal stem cells
Source: Stem Cell Res Ther. 2020 Jun 9;11:226. doi: 10.1186/s13287-020-01744-1 (PMC7285747; doi:10.1186/s13287-020-01744-1)

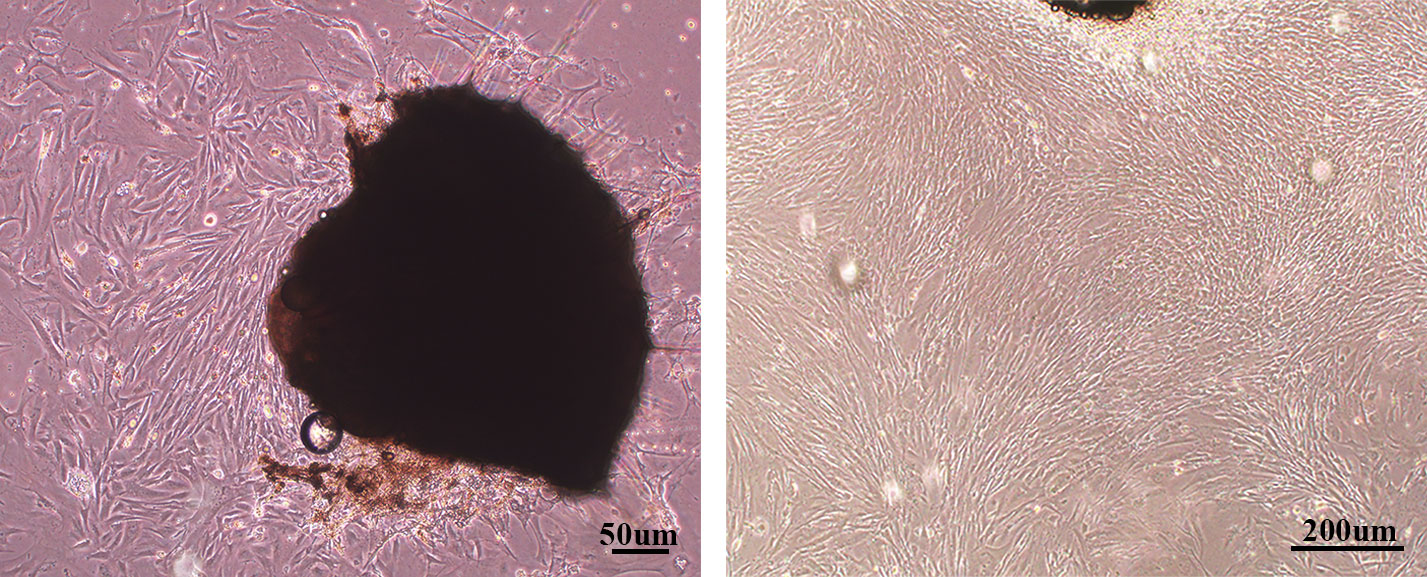

Supplement: Supplementary file 1 — Additional file 1: Fig. S1. ADMSCs exhibit fibroblast-like, spindle-shaped morphology, were spiral shaped and in alignment. [file 13287_2020_1744_MOESM1_ESM.tif]

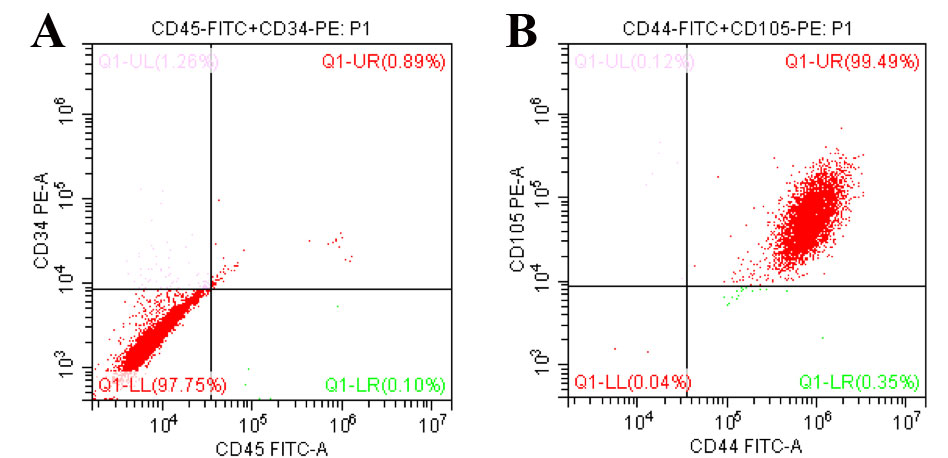

Supplement: Supplementary file 2 — Additional file 2: Fig. S2. Flow cytometry analysis showing the presence of CD34 and CD45 negative (0.89%) surface markers (a), and CD44 and CD105 positive (99.49%) surface markers (b). [file 13287_2020_1744_MOESM2_ESM.tif]

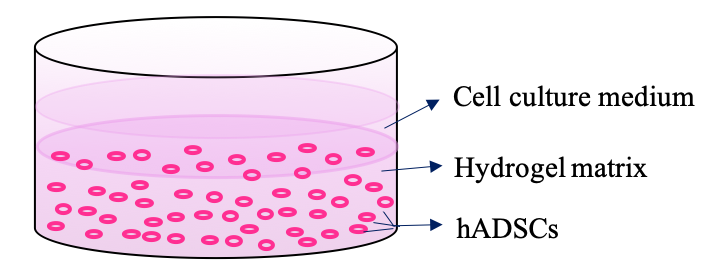

Supplement: Supplementary file 3 — Additional file 3: Fig. S3. Model graph of 3D hydrogel culture. [file 13287_2020_1744_MOESM3_ESM.tif]
